# Lower-Third Standardized Letters of Evaluation in Emergency Medicine: Does Gender Make a Difference in Match Outcome?

**DOI:** 10.7759/cureus.19507

**Published:** 2021-11-12

**Authors:** Erica B Shaver, Haley D Frauen, Rachel Z Polinski, Stephen M Davis, Kimberly D Quedado, Joseph Hansroth, Kristin H Davis, Michelle R Angeline, Christopher S Kiefer

**Affiliations:** 1 Emergency Medicine, West Virginia University School of Medicine, Morgantown, USA; 2 Health Policy and Leadership, West Virginia University School of Public Health, Morgantown, USA

**Keywords:** medical education, residency selection, gender bias, emergency medicine, student assessment

## Abstract

Objective

The purpose of this study was to determine whether gender influences the likelihood of receiving a lower-third global assessment (GA) on the standardized letter of evaluation (SLOE) submitted as part of the emergency medicine (EM) application process as well as the impact of gender on ultimate match outcomes for applicants receiving a lower-third GA ranking. Our hypothesis was that female applicants with a lower-third GA ranking have a higher risk of not matching.

Methods

We conducted a retrospective cohort study evaluating U.S.-based allopathic applicants to a single EM residency program in the Mid-Atlantic region during the 2017-2018 and 2018-2019 match cycles. GA SLOE rankings and gender for all applicants were extracted and compared to the National Resident Matching Program (NRMP) data for each applicant on match outcome. Comparative analyses were conducted between gender and SLOE GA rankings in order to obtain an odds ratio (OR) of gender and match outcomes.

Results

A total of 2,017 SLOEs were reviewed from 798 applicants in the 2018 and 2019 EM match cycles. Overall, 716 (90%) applicants successfully matched in EM, with 82 (10%) applicants failing to match into EM; 277 students had at least one lower-third GA ranking. For all applicants, having at least one lower-third GA ranking was associated with a significant risk of not matching (OR: 0.20; 95% CI: 0.12-0.34). Of the 277 students with at least one lower-third GA ranking, 85 (31%) were female and 192 (69%) were male. Of the female applicants with a lower-third GA ranking, 15 (18%) failed to match in EM, and 39 (20%) of the males failed to match in EM. For applicants with a lower-third GA ranking, female gender alone was *not *associated with a significantly increased risk of not matching (OR: 1.18; 95% CI: 0.61-2.21).

Conclusions

Female applicants receive a lower-third GA ranking less frequently than their male counterparts. One or more lower-third rankings on the GA significantly reduced an applicant’s chances of matching into an EM program. For those with a lower-third GA ranking, female gender alone does not significantly increase the risk of not matching into EM.

## Introduction

Residency program directors (PDs) in every specialty must filter through large amounts of data points when selecting applicants for residency interviews and ultimately making decisions on ranking those applicants. In emergency medicine (EM), it is well known that the standardized letter of evaluation (SLOE) serves as one of the most important, if not the most important, parts of the application for PD reference when making these decisions [1,2]. Prior work has repeatedly demonstrated the SLOE to be the primary driver in the application review process. This reputation likely comes from the fact that the SLOE is a standardized template form for evaluation that is easy for authors to complete, provides objective information that is pertinent to EM as a specialty, and is succinct to allow for efficient review by program leaders [2,3].

The SLOE, as introduced above, provides the opportunity for authors to provide objective and comparative information on applicants in EM. The letter consists of sections on background information, applicant qualifications for EM (compared to other EM applicants), a two-part global assessment (GA) section, and, finally, a written comments and narrative section. The SLOE template allows the author to assess an applicant’s performance in a way that can be easily compared to other EM-bound applicants in a construct that is not onerous for authors to complete and easy for letter readers to review and interpret.

The GA section of the SLOE asks the author to compare the applicant’s performance to all other EM-bound applicants in the past year and estimate what position the applicant will fall into on their program rank list. Prior work has suggested that it is vital to differentiate applicants by utilizing the entirety of the SLOE ranking scale [4]. The intent of the “thirds” in the GA portion of the SLOE is to evenly distribute applicants for EM across these thirds, understanding that, in theory, there will be many suitable applicants for EM that find themselves with a lower-third ranking. Paradoxically, there has been some concern that applicants receiving any lower-third GA ranking are less likely to match. Given that PDs are often part of the SLOE authorship group and may find themselves in the dual role of student advisor and resident recruiter, they could be concerned that placing applicants in the lower-third on either of the SLOE GA assessment questions is the “kiss of death”. As such, and despite the guidance to distribute applicants evenly across the thirds, only 10-12% of applicants receive a lower-third ranking on any GA question [4]. Although a large percentage of applicants with a lower-third ranking will ultimately match into the specialty, prior work has confirmed the inferred concern that applicants with any lower-third GA ranking are indeed at an increased risk of not successfully matching in EM [5].

Gender bias in academic medicine is well established. Female faculty physicians are less likely to be promoted to associate or full professor appointments and are less likely to achieve leadership positions within their departments, with only 16% of academic department chairs being female [6]. In the rare occasion that they do secure the same leadership roles as their male counterparts, they make, on average, $20,000 less in compensation [7]. Not unexpectedly, the infrequency of female physician presence in academic EM has become a self-fulfilling prophecy. Female EM residents are also a minority of the overall EM resident pool, comprising approximately 38% and 34.9% of EM residents in 2013 and 2017, respectively [7,8]. Similarly, only 27% of academic EM faculty in the country are female [7], suggesting that the specialty is not successfully recruiting or positioning female physicians for success in large numbers at the current time.

Limited prior work on the SLOE and gender has demonstrated two themes. First, female students outperform their male counterparts regarding composite scores and rank list positions. Secondly, authors use more words to describe female applicants, leading to slightly longer (on average 17 more words) narratives for female applicants. Females are more frequently described with words such as “social” (friendliness, interpersonal ability assessments) and “ability” (talent, intelligence, insight, creativeness) [10].

Given that the SLOE is the most important part of an EM application and some preliminary work has demonstrated potential variance in SLOE content based on gender, there is an opportunity and need to describe the real-world outcomes of female applicants receiving a lower-third SLOE GA ranking [5]. The primary objective of this study was to determine whether or not female applicants receiving a lower-third GA ranking were more likely to not match than their male counterparts receiving a lower-third GA ranking.

## Materials and methods

This study is a follow-up study to analyze the impact of lower-third SLOE GA rankings on applicant match outcome status, with respect to gender. It utilizes the same data set as a previously conducted retrospective cohort study assessing the impact of any lower-third GA rankings on match status of all EM applicants, regardless of gender [5]. This follow-up, retrospective cohort study includes analysis of all U.S. allopathic applicants to a single EM residency program during the 2018 and 2019 match cycles. The study site has participated regularly in the National Resident Matching Program (NRMP) match since 1993, and for its 10 residency positions per year, it receives applications from all regions of the USA. averaging 750 applications per year. U.S. allopathic applications make up over half of the study sites’ total applications, making it consistent with the national average of 59-64% of applicants being allopathic in the 2018-2019 match cycles. Our study sample included 32.9 % female applicants, which mirrors the overall number of female physicians in EM (28%) as reported by the American Association of Medical Colleges (AAMC) [11].

All applicants from Liaison Committee on Medical Education (LCME) schools who had applied to the study site via the Electronic Residency Application Service (ERAS) during the 2017-2018 and 2018-2019 residency application cycles were reviewed by five abstractors (J.H., K.D., R.P., H.F., M.A.). Applicants were excluded from the analysis if they were (1) non-U.S. allopathic applicants or (2) U.S. allopathic applicants without a SLOE in their application or (3) if gender was not able to be determined from application review. SLOE rankings were obtained from the standard information within each applicant’s ERAS file. The abstractors analyzed all SLOEs submitted as part of the standard application process.

Each SLOE was reviewed for the following two GA questions: (1) Compared to other EM residency candidates you have recommended in the last academic year, this candidate is in the top 10%, upper-third, middle-third, or lower-third? and (2) How highly would you estimate the candidate will reside on your rank list? (with the same response options as question 1, with the addition of “unlikely to be on our rank list”). SLOEs from EM subspecialty rotations (i.e. pediatric EM, ultrasound, toxicology, etc,) were included. The number of lower-third rankings in either GA response in all available SLOEs was recorded, with no distinction being made between whether the lower-third ranking(s) was assigned within a single SLOE or multiple SLOEs for each applicant. Once we had tabulated applications based on the presence/absence of a lower-third GA ranking, we further categorized them on the basis of gender (i.e. male applicants with any lower-third GA ranking and female applicants with any lower-third GA ranking).

Final match status was determined utilizing one of two methods. For applicants who were interviewed and subsequently placed on the study site’s final rank order list (ROL), the abstractors determined final match status by utilizing the “Match Results by Ranked Applicant” reported by NRMP at the conclusion of the match cycle for the two study years. For applicants who were not invited for interview or who were interviewed but not placed on the study site’s ROL, the residency leadership (E.S., C.K.) used the AAMC identification number to query the applicant’s match outcome in the NRMP database (available to institutional officials, PDs, and program coordinators).

Descriptive statistics were calculated for all variables. The degree to which the distribution of continuous variables departed from normality was assessed using the Shapiro-Wilk W test, with significant (p < 0.05) results indicative of a non-normal distribution. Nonparametric tests were conducted in the presence of non-normal outcome distributions.

Contingency table analysis was employed to explore the association between applicants with a lower-third GA ranking, with respect to gender and match status, with outcomes expressed as odds of matching into EM, and the presence of any lower-third GA ranking on a SLOE, for both male and female applicants, with outcomes expressed as odds ratios (ORs). We calculated 95% confidence intervals (CIs) around each OR estimate. Ratios that did not include 1 in the confidence interval were considered to be statistically significant associations.

The study protocol was reviewed and approved by the Institutional Review Board at the study site, with a waiver of informed consent.

## Results

During the 2018-2019 match cycles, a total of 1,405 EM applicants applied to the EM residency program at the study site. Of those 1,405 applicants, 798 applicants were from U.S. allopathic institutions. The study sites cohort of U.S. allopathic applicants represented 22% of all U.S. allopathic applicants applying to EM during the two match cycles analyzed [12,13]. Of the total 798 applicants, 17 (2%) had no SLOEs for review in their ERAS applications and, therefore, were excluded from analysis. A total of 2,017 SLOEs were reviewed from the remaining 781 applicants in the cohort. The SLOES reviewed in the applicant cohort were generated from 190 EM residency programs across the country, representing 76.9% of the total EM residency programs in the USA, at the time of the study. The demographic characteristics of the applicants, their respective medical schools, and the institutions authoring their SLOEs are included in Table 1.

**Table 1:** Demographic characteristics of all applicants in the study cohort. This table was originally published as part of an article published by this author group exploring the impact of lower-third GA ranking on all applicants, regardless of gender [5]. Regions in this figure and in the remainder of the paper are derived from the Society for Academic Emergency Medicine residency directory: https://member.saem.org/SAEMIMIS/SAEM_Directories/ResidencyMap/SAEM_Directories/P/ResidencyMap.aspx?hkey=1e134970-ec57-4862-87fb-6971bad7a77b CK, clinical knowledge; GA, global assessment; LCME, Liaison Committee on Medical Education; SD, standard deviation; SLOE, standardized letter of evaluation; USMLE, United States Medical Licensing Examination

Applicant demographics	
Total number of applicants reviewed	781
Male, n (%)	524 (67.1%)
Female, n (%)	257 (32.9%)
USMLE Step 1, mean (SD)	222 (±16)
USMLE Step 2 CK, mean (SD)	239 (±14)
Total unique medical schools, n (% of total LCME institutions)	126 (81.2%)
Medical school region, n (% of sample size)	
New England	53 (6.8%)
Mid-Atlantic	96 (12.2%)
South Central	93 (11.9%)
Southeastern	203 (25.9%)
Midwest	159 (20.3%)
Great Plains	96 (12.2%)
Western	81 (10.3%)
Medical school type, n (% of sample size)	
Public	595 (76.1%)
Private	186 (23.8%)
Demographics of institutions providing SLOEs	
Total number of SLOEs reviewed	2017
Total residency programs represented, n (%)	190 (76.9%)
SLOE program region	
New England	210 (10.8%)
Mid-Atlantic	272 (14.0%)
South Central	207 (10.7%)
Southeastern	523 (27.0%)
Midwest	328 (16.9%)
Great Plains	197 (10.2%)
Western	202 (10.4%)
SLOE program type, n (%)	
University	1372 (68.0%)
Community	408 (20.2%)
County	99 (4.9%)
No residency program at site	50 (2.5%)
Military	10 (0.49%)
Program data unavailable	78 (3.8%)

Overall, 703 (90%) applicants successfully matched in EM, with 78 applicants (10%) failing to match. Of those 703 students, 277 had at least one lower-third GA ranking. For these 277 applicants, having at least one lower-third GA ranking significantly decreased the odds of a successful match by 79% (OR: 0.21; 95% CI: 0.12-0.34) (Table 2). Female applicants in our study population received fewer SLOEs containing any lower-third GA rankings compared to their male counterparts. In regard to gender and any lower-third GA rankings, which was the primary focus of this subset analysis, of the 277 total applicants with any lower-third GA ranking, 85 (31%) were female and 192 (69%) male. Females with any lower-third GA ranking failed to match 18% of the time, similar to their male counterparts with any lower-third GA ranking who failed to match 20% of the time.

**Table 2:** Odds of matching into an emergency medicine residency for applicants with no lower-third GA ranking and those with any lower-third GA ranking. GA, global assessment

	Applicants who did match (%)	Applicants who did not match (%)	Odds ratio (95% CI)
Overall	703 (90.0%)	78 (10.0%)	
No lower-third GA	480 (95.2%)	24 (4.7%)	4.84 (2.91-8.03)
Any lower-third GA	223 (80%)	54 (20%)	0.21 (0.12-0.34)

Within their own genders, both female and male applicants with any lower-third GA ranking had an increased risk of not matching when compared to their same gender applicants without any lower-third GA ranking (female OR: 0.13 (95% CI: 0.04-0.39); male OR: 0.23 (95% CI: 0.13-0.43)] (Table 3).

**Table 3:** Gender-matched odds of matching into emergency medicine for both females and males with and without a lower-third GA ranking. GA, global assessment

	Applicants who did match (%)	Applicants who did not match (%)	Total	Odds ratio (95% CI)
Female applicants				
No lower-third GA	167 (97%)	5 (3%)	172	0.13 (0.04-0.39)
Any lower-third GA	70 (82%)	15 (18%)	85	
Male applicants				
No lower-third GA	313 (94%)	19 (6%)	332	0.23 (0.13-0.43)
Any lower-third GA	153 (80%)	39 (20%)	192	

When comparing specifically between female and male applicants with any lower-third GA ranking, which was the primary study outcome, female gender alone was not associated with a significantly increased risk of not matching (OR: 1.18; 95% CI: 0.61-2.21) (Table 4).

**Table 4:** Comparison of match rates into emergency medicine among male and female applicants with any lower-third GA ranking. GA, global assessment

	Applicants who did match (%)	Applicants who did not match (%)	Odds ratio (95% CI)
Females with any lower-third GA	70 (82%)	15 (18%)	1.18 (0.62-2.29)
Males with any lower-third GA	153 (80%)	39 (20%)	

## Discussion

In the current environment that we are faced with in medical education, it is imperative for all medical educators to be aware of potential implicit gender biases that may color their evaluation of residency applicants. The potential for gender bias in medical learner evaluation has been previously described in a variety of settings, from literature suggesting that nursing evaluations tend to be harder on female residents compared to male resident colleagues [14] to studies showing that attending EM physicians traditionally value male resident characteristics such as decisiveness, increased confidence, and assertiveness [15]. Given this prior literature that suggests there is likely a gender bias present in the evaluation of medical learners, it was somewhat surprising that our study showed no gender-specific differences in EM match rates when comparing males and females with any lower-third GA rankings.

In some ways, our findings mirror those of Andrusaitis et al., who suggested that female medical students outperform their male counterparts in EM clerkships, as measured by the SLOE [9]. Our study findings suggest that a smaller proportion of female applicants received a lower-third GA ranking compared to male counterparts pursuing the same specialty. Given the findings from our primary study’s larger data set [5], we know that an applicant, regardless of gender, with any lower-third GA ranking is at a significantly increased odds of not matching into EM. Therefore, the decreased frequency with which females receive a lower-third GA ranking on the SLOE seems to suggest that females are actually not discriminated against and are perhaps actually even better positioned for a successful match in EM when compared to their male counterparts.

To be transparent, we are uncertain of the “why” behind our results, suggesting there is no gender bias against females receiving any lower-third GA ranking, as demonstrated by the fact that there is no significant difference in EM match rates between females and males receiving a lower-third GA ranking. Although our study was not designed to include qualitative analysis of the narrative commentary of the SLOE, and we cannot directly draw any conclusions regarding the professional and personal traits that may protect female EM applicants from receiving a lower-third GA ranking compared to their male counterparts, prior studies have shown that SLOE authors describe women more frequently with words such as “ability” (i.e. talented, brilliant, adept, etc.) and “social” (i.e. caring, empathetic, bright, etc.) [10].

As we (C.K. and E.S.) have evolved as residency program leaders and historically identified personal and professional characteristics that ultimately make an EM resident successful during training, we also recognize that we perhaps value different characteristics in residents during different stages of their training. When evaluating fourth-year medical school applicants for matriculation into an EM residency program, we are likely to value candidates who are kind, humble, intellectually curious, and open to feedback, and will be well liked by their colleagues and serve as ambassadors for the program they represent. It is likely at that stage in the resident evaluation/selection process that we place higher value upon the “social” and “ability” characteristics as cited earlier [10]. However, as a resident trainee approaches graduation from their training program, evaluators likely value different characteristics when assessing them, valuing qualities such as decisiveness, autonomy, and the ability to lead with confidence-traits that are more traditionally atttibuted to the male gender [15].

Given the disparities between traits that are valued in fourth-year medical students applying to an EM residency compared to those valued in later stages of training, perhaps it is not surprising that the value that program leaders may be placing on the above highlighted “social” and “ability” characteristics at that early stage in training may be protecting the female applicant from receiving a lower-third GA ranking in their SLOE when compared to their male counterparts. Further qualitative work looking specifically at SLOE narratives may help identify specific descriptive terms that portend which female and male applicants may receive a lower-third GA ranking.

Potential limitations of this study include the analysis of applicants to a single residency program in the Mid-Atlantic region of the USA, which may limit the generalizability of the results, although demographics suggested that we had a representative sample from 22% of all U.S. allopathic applicants and roughly equivalent representation from medical schools, with SLOE authors from all around the country. An additional limitation is that this study was conducted before the completion of the ACGME-AOA (Association of American Colleges of Osteopathic Medicine-American Osteopathic Association) merger at a program historically focused on allopathic student recruitment. The student population of study may limit the generalizability of the results to other student populations, such as osteopathic or international medical graduates. Future studies could take on a multi-institutional approach and/or include additional student groups to further validate these results. Lastly, we did not include an analysis of which performance factors placed applicants at a risk of obtaining a lower-third GA ranking. Surveying SLOE authors to obtain this information may be useful in future explorations of this topic.

## Conclusions

Our study evaluated the effect of gender influence on the likelihood of receiving a lower-third GA ranking on the EM SLOE and the impact of the lower-third GA ranking of U.S. allopathic female applicants successfully matching when compared to their U.S. allopathic male counterparts, also receiving a similar lower-third GA ranking. All U.S. allopathic EM applicants, irrespective of gender, who receive any lower-third GA ranking are at an increased risk of not matching into the specialty. More specifically, U.S. allopathic female applicants receive a lower-third GA ranking less frequently than their U.S. allopathic male counterparts. For those U.S. allopathic applicants with any lower-third GA ranking, female gender alone does not significantly increase the risk of not matching.
